# Diversity of Forest Litter-Inhabiting Ants Along Elevations in the Wayanad Region of the Western Ghats

**DOI:** 10.1673/031.008.6901

**Published:** 2008-11-12

**Authors:** Thomas K. Sabu, P. J. Vineesh, K.V. Vinod

**Affiliations:** Litter Entomology Research Unit, PG and Research Department of Zoology, St. Joseph's College, Devagiri, Calicut, Kerala, India 673008

**Keywords:** mid-elevation abundance, abiotic and biotic factors, South Western Ghats forests, South India

## Abstract

Litter ant diversity and abundance in relation to biotic and abiotic factors were analyzed at five primary forest sites lying between 300 to 1650 meter above mean sea level in the Wayanad region of the Western Ghats in Kerala, southern India. Ant abundance and species richness peaked at mid-elevations influenced by the presence of favourable physical conditions and abundance of prey resources. Dominance of ants preferring termites and Collembola as prey at sites rich in their specific prey resources indicate the influence of local prey resource availability in determining ant distribution. Dominant species (*Tapinoma* sp. and *Solenopsis* sp.) had wider distributions, being present at all elevations. Physical factors (slope of the terrain, rainfall, moisture, humidity, temperature) and prey resource availability (insect larvae, termites, Collembola) influenced ant species abundance at a regional scale, whereas at local scales, site specific variations in the relationship between abundance of ants and prey-predators and physical factors were recorded. The present study highlights the need to consider site-specific abiotic and biotic factors while examining the distribution patterns of litter ants along altitudinal gradients in other regions of the Western Ghats, which is a recognised hot spot of biodiversity with wide regional variation in vegetation types and faunal distribution patterns.

## Introduction

Information on biogeographical variation in species richness and endemic richness is critical to understanding and conservation of biological diversity, and to develop rigorous conservation plans for a region (Fisher and Robertson 2002; Grytnes and Vetaas 2002; Fu et al. 2004, 2006). Most research to date has focused on providing vertebrate data for conservation assessment, and for many groups of invertebrates we lack even basic information in tropical ecosystems (Fisher and Robertson 2002). This is particularly true for the litter ant fauna of subtropical Western Ghats forests, a recognized global hot spot of biodiversity in India (Myers et al. 2000; Myers 2003; Bossuyt et al. 2004) for which spatial distribution patterns and the factors that influence species richness and abundance along environmental gradients such as elevations are not available. Two basic types of elevational diversity patterns of litter ants have been documented from Afrotropical regions with, 1) a steady decline in diversity and abundance from low to high elevations in Venezuela (Janzen et al. 1976), Costa Rica (Janzen 1973), Panama (Olson 1994) and Malaysia (Brühl et al. 1998) and, 2) a mid-elevational rise in ant diversity with low abundance at lower and higher elevations from Philippines (Samson et al. 1997) and Madagascar (Fisher 1996, 1998, 1999a; Fisher and Robertson 2002). The present study provides information on how the diversity of forest litter ants varies with elevation along the altitudinal gradients of Wayanad region of the Western Ghats.

Further, elevation is merely a surrogate for a suite of biotic and abiotic factors that influence species richness (Rahbek 1995). Therefore, identifying ecologically meaningful causal factors is essential in order to explain variation in species richness along elevational gradients (Naniwadekar and Karthikeyan 2007). Hence, we aim to ascertain which habitat specific biotic and abiotic factors are most important in affecting the distribution of litter ants along different elevations in the moist windward region of the Western Ghats.

Factors such as litter temperature, humidity, litter depth, rainfall, and slope of the terrain have been shown to be influencing abundance and elevational distribution of litter ants elsewhere (Samson et al. 1997; Brühl et al. 1999; Kaspari et al. 2000; Fisher and Roberstson 2002; Sanders et al. 2003; Vineesh et al. 2007). These factors become more extreme with increasing elevation and are responsible for the absolute limit of ant occurrence in tropical rain forests (Leideritz 1993; Brhul et al. 1998). Prey resource availability (coleopteran and dipteran larvae) and ant competitor predators (spiders, carabids) have often been identified as biotic factors controlling the variation in ant abundance along elevations in tropical forests (Darlington 1971; Janzen 1983; Hölldobler and Wilson 1990; Stork and Brendell 1990; Olson 1994; Brühl et al. 1998). Reduced competition from ants in montane leaf litter is suggested as leading to the ecological replacement of ants by carabids and spiders (Olson 1994). An exponential decrease of ant species with elevation has been linked to the reduction of food resources for ants viz., Collembola, termites and ants themselves (Brühl et al. 1998). Furthermore, studies on the altitudinal variation of litter insects along the elevations of the Wayanad region of Western Ghats (Sabu 2005) revealed an overall high abundance in mid-elevation, which includes many potential prey resources of litter ants. However no data are available on the influence of the above stated factors on litter ant diversity from any region of the Western Ghats. The broad objectives of the present work are to, 1) assess the general pattern of diversity of litter ants and, 2) to identify the potential abiotic and biotic factors that affect their diversity along the elevations of the Wayanad region of Western Ghats.

## Materials and Methods

### Study region

**Figure 1.**
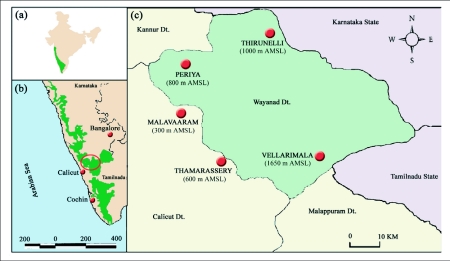
(a) Map of India showing the location of the Western Ghats, (b) the Western Ghats and (c) Wayanad forests and location of study sites.

Study sites were located at Malavaram (300 m AMSL), Thamarassery (600 m AMSL), Periya (800 m AMSL), Thirunelli (1000 m AMSL) and Vellarimala (1650 m AMSL) (Figure 1). Wayanad is an east sloping, gently undulating, medium elevational plateau lying within an altitudinal range of 300–1650 m AMSL (11°58′N – 11°30′N; 75°45 – 76°28′E). The plateau abruptly descends into the Malabar plains in the West and merges with the Mysore plateau in the East. It is a transition area between the moist forests of southern parts of the Western Ghats and the dry-deciduous forests of south Deccan Plateau, including faunal and floral elements species from both, leading to high species richness (Rodgers and Panwar 1988; Pascal 1991; Nair 1991). The northeast monsoon from September to October supplements the June to July southwest monsoon rainfall. Moistureladen southwest monsoon winds sweep in from the Malabar Coast and rise above the mountain range, releasing more than 2,500 mm of rainfall. However because of the deeply dissected topography some areas receive more than 6,000 mm of rainfall (Forests and Wildlife Department working plan 2001a, b).

### Sampling

Diversity and abundance of litter-inhabiting ant communities were analysed using Winkler sifting methods (Besuchet et al. 1987) and other insect groups (prey and predator) using Berlese - Tullgren funnel methods (Schillhammer 2001). Litter collections were not undertaken for at least four days after rain to avoid sticking of ants to litter material, since the wet forest floor may discourage foraging by ants (Brühl et al. 1998). Sampling was performed at each altitudinal site in August after the southwest monsoon period and in November after the northeast monsoon period in 2002. Litter ants were sampled following the widely recognized standardized protocol “Ants of the Leaf Litter” (Alonso and Agosti 2000) that allows quantitative comparisons between litter ant communities in space and time (Alonso and Agosti 2000; Longino et al. 2002; Leponce et al. 2004). As per this method, a single-line transect (200 m) and a sampling interval of 10 m (20 samples) followed by Winkler extraction for 24 hrs is the minimum sampling effort necessary for characterizing the leaf-litter ant assemblages (Leponce et al. 2004). Two parallel preliminary line transects of 150 m (one for Winkler sifting and the other for Berlese-Tullgren funnels extraction) separated by 10 m was constructed in each altitudinal site. Each litter sample was collected by placing a ¼ m^2^ wooden quadrat frame on to the litter and scraping up all litter and loose humus from within the frame area into a large polythene bag. Samples were collected as quickly as possible to prevent escape of animals. Two sets of 30 replicate samples (15 samples after the southwest monsoon period and 15 after the northeast monsoon period) from each site were available for Winkler sifting of ants and Berlese-Tullgren funnel extraction of litter fauna. Samples for the extraction of ants were kept undisturbed in the polythene bags for 48 hrs in the shade, sifted into a white plastic sheet of 3 × 3 m, and ants were hand picked and transferred to 70% alcohol in labelled containers. Ants were identified using Bolton (1994) and Bingham (1903). Specimens were further compared with the collections at the museums of St. Xavier's College, Alwaye, and the University of Calicut. Voucher specimens have been temporarily deposited at the museum of St. Xavier's College, Alwaye.

**Figure 2.**
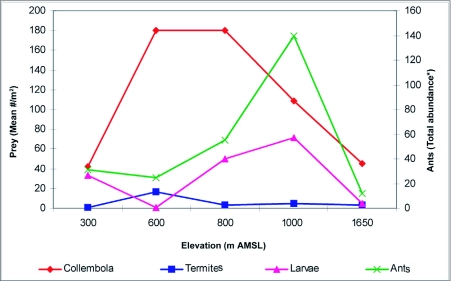
Overall abundance of litter ants from 30 samples and prey resources along the varying elevations of Wayanad forests.

**Figure 3.**
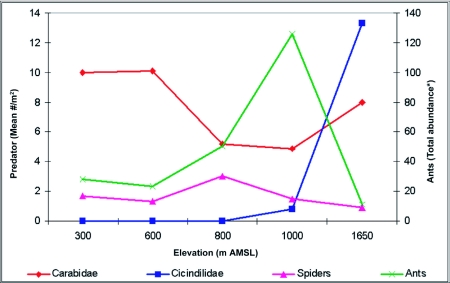
Overall distribution of ants from 30 samples and competitive predators at each elevation.

Litter samples were brought to the research station and fauna was extracted using a Berlese-Tullgren funnel apparatus (30.5 cm diameter, 35.6 cm height, 4–6 mm mesh screen, 25 W bulb) for 1–2 days into 70% alcohol. Extracted fauna was separated, identified up to the morphospecies level, counted and the abundance of ant prey resources and ant competitors at each site was recorded (Figures 2 and 3). Altogether, ants and other litter arthropod fauna present within 150 litter samples were available for data analysis.

Physical and climatic characteristics of the sites are included in Table 1. Forest vegetation type is based on the working plan of the Kerala State Forest and Wildlife Department. Rainfall data was collected from the weather stations of the Forest Department at Thirunelli, Harison's Malayalam Plantation, Arapetta Estate at Meppadi, Irrigation department at Peruvannamoozhi, KSEB Divisional Office at Periya and Taluk Office at Vythiri (Revenue Department). Humidity and temperature were determined using a thermo-hygrometer. The slope of the terrain at each site was calculated using the trigonometric formula ‘tan θ;’ (where, ‘θ’ is the angle of inclination) Jacobs and Mayer 1972). Litter depth was determined by measuring depth at 30 spots per plot with a standard 30 cm ruler. Humidity, temperature, slope of the terrain and litter depth were calculated as the mean of 30 sampling spots at each site.

**Table 1.**
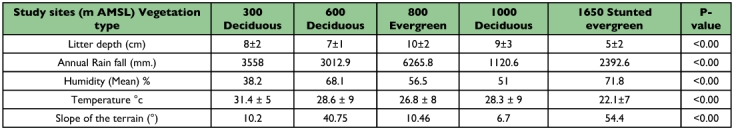
Physical characteristics of the study sites in the Wayanad region of the Western Ghats.

### Data analysis

For analysis of community diversity of colonial organisms such as ants the frequency of occurrence of species in each sample is taken into account and not the number of each individual species encountered during the inventory, as a single sample may contain an extreme abundance of a rare species or a whole nest was collected. Although non-quantitative in terms of numbers of individuals, occurrence records at least provide an estimate of encounter with the various species, and this procedure is commonly suggested to evaluate the diversity of leaf-litter ants (Longino 2000; Brühl et al. 2003; Pfeiffer et al. 2003). Hence, to get an estimate of the abundance of ant species we used incidence of species in samples as the measure of abundance. Ant species present in all 30 samples of a site were given the abundance value as 30 and those present in only 1 sample as 1. Site-wise community diversity was analyzed with the non parametric Shannon's diversity index (H') (Shannon and Weaver 1949; Bruhl et al. 2003; Maguran 2004). Affinities between the ant assemblages along the elevations were analysed with Sorensen's index, Jaccard's index (Magurran 2004) and Trellis diagram (Vu and Nguyen 2000).

Statistical significance of relationships between ant abundance at each elevation, using the mid-elevation study site as a standard, was done using Dunnett's test, which is a non-parametric test for comparing the mean of each group with control mean (Mikola et al. 2001; Zar 2003; Clay et al. 2005). The Spearman rank correlation was used instead of Pearson's correlation to analyse the relationship between abundance of ants and prey-predators (Zar 2003) as sampling methodology differed for both groups. The presence of spatial autocorrelation in species richness of ants and climatic factors were checked with Moran's I index (Rangel et al. 2006). The influence of habitat physical factors (rainfall, temperature, litter depth, humidity, and slope of the terrain) on ant abundance both at regional (5 elevations together) and local (individual elevation) levels was examined with multiple regression (Zar 2003). Multicollinearity among the variables was analyzed with auxiliary regression (Gujarathi 2003) and exclusion of collinear variables was done based on F value (Graham 2003; and personal communications, MH Graham). Rainfall was not considered at local scales due to lack of sufficient data. The influence of elevation on ant abundance was not analysed, as the study points were not on a single altitudinal gradient.

Alpha and Beta diversity was computed with the program Estimates (Ver.6.0b.1) (Colwell 2000). Correlation and regression analyses were done with Megastat version 10.0 (Orris 2005) and Dunnett's test with Ky Plot software (Yoshioka 2000). Spatial autocorrelation (Moran's I) was estimated with SAM v2.0 (Rangel et al. 2006).

## Results

**Figure 4.**
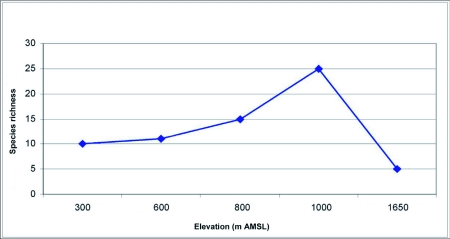
Hump-shaped richness pattern of litter ants along the varying elevations of Wayanad forests.

**Figure 5.**
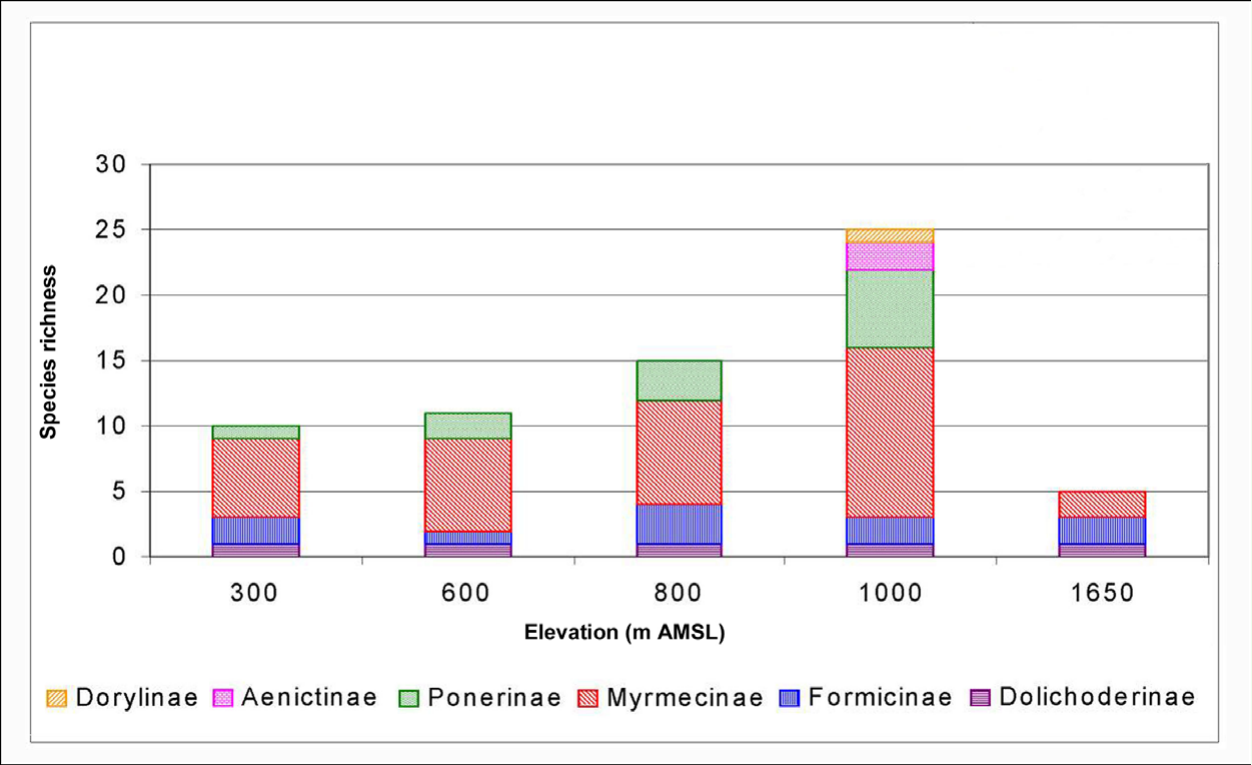
Family-wise species richness of litter ants along the varying elevations of Wayanad forests.

**Figure 6.**
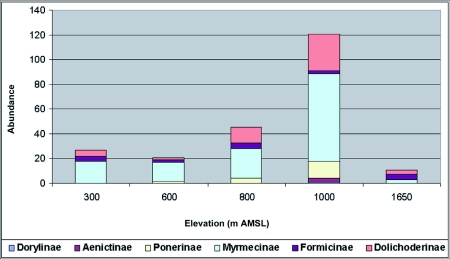
Family-wise abundance of litter ants along the varying elevations of Wayanad forests.

**Figure 7.**
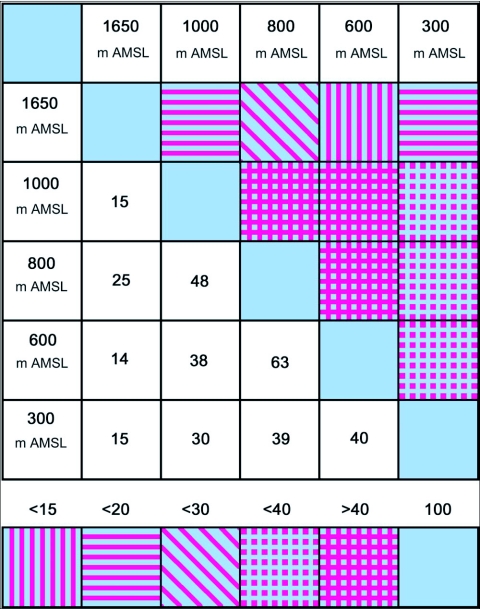
Trellis diagram showing the affinities between litter ant assemblages at five different elevations in the Wayanad forests.

Ant (species turn over) species richness increased from 300 m AMSL to 1000 m AMSL elevations and subsequently decreased recording a hump-shaped peak in mid-elevations (Figure 4). Twenty-nine ant species belonging to 18 genera under 6 subfamilies were reported in total. Myrmecinae followed by Ponerinae were the most common and species rich family from 300 to 1000 m AMSL sites. The Ponerinae/Myrmecinae ratio was 0.17 at 300 m AMSL, 0.25 at 600 m AMSL, 0.38 at 800 m AMSL, and 0.46 at 1000 m AMSL. At 1650 m AMSL, *Formicinae* followed by Myrmecinae (ratio of 0.5) dominated and Ponerinae was not recorded. Eleven species were restricted to specific elevations (Table 2). *Tapinoma* sp and *Solenopsis* sp.2 were the dominant taxa and were present at all elevations. Species richness was greatest at 1000 m AMSL (25 species) and lowest at 1650 m AMSL (5 species). Myrmecinae was the most abundant and species rich family at all elevations except in Formicinae dominated higher elevations (Table 2). Members of Dorylinae and Aenictinae were recorded only at 1000 m AMSL, and Ponerinae, Dorylinae and Aenictinae were not recorded in the higher elevations at 1650 m AMSL (Figure 5). The highest Shannon diversity was recorded at 1000 m AMSL and lowest at 300 m AMSL and 1650 m AMSL (Table 2). Dunnett's test revealed significant variation in the ant abundance between 1000 m AMSL and other altitudinal sites. The highest variation was between 1650 m AMSL (P < 0.001) and 600 m AMSL (P < 0.001) sites, and lowest between 800 m AMSL (P < 0.05) and 300 m AMSL (P < 0.01) sites. The highest faunal similarity was recorded between 600–800 and 800–1000 m AMSL and the lowest between 600–1650 m AMSL sites (Table 4, Figures 6 and 7).

Collembola and termites were abundant at the 600 m AMSL site, and Collembola and insect larva at the 300, 800, 1000 and 1650 m AMSL sites. Carabids were abundant at all elevations except at the 1650 m AMSL site where Cicindelidae dominated (Figures 2 and 3).

Spearman rank correlation analysis between abundance of prey resources and ants at a regional scale showed that insect larval populations (ρ = 0.687; P < 0.001) and Collembola (ρ = 0.358; P < 0.001) had significant positive correlations with ant abundance, and no correlation with termite population (ρ = 0.09; P > 0.05). Correlation analysis between the abundance of ants and competitive predators *viz*., spiders showed significant positive correlation (ρ = + 0.39; P < 0.001), Carabidae showed significant negative correlation (ρ = -0.23; P < 0.01) and Cicindelidae showed insignificant negative correlation (ρ = -0.04; P > 0.05). Correlation analysis showed local scale (altitude specific) variations in the relationship between abundance of ants and prey-predators. Significant positive relationship was found with larvae at all elevations (Table 4) with Collembola except at the 600 m AMSL site; with spiders except at the 600 and 800 m AMSL sites, with Carabidae and termites only at the 300 m AMSL site, and a negative relationship with Cicindelidae at the 1000 and 1650 m AMSL sites.

The absence of spatial autocorrelation using the species richness (Moran's I = 0.048; P = 0.451) and physical parameters was revealed (Table 5). Multiple regression showed significant influence of physical factors on ant abundance at the regional level (R^2^ = 64.3, F_5_, 144 = 51.94, P < 0.001). Elevation-wise analysis showed that physical factors significantly influenced ant abundance except at 800 m AMSL (Table 6). Analysis of the relationship between abundance of ants and individual explanatory variables revealed the presence of significant multicollinearity between variables at all sites. Further analysis after exclusion of multicollinear variables showed significant influences on ant abundance at 1650 m AMSL for litter depth and slope of the terrain, at 1000 m AMSL for litter depth and humidity, at 600 m AMSL for litter depth, and at 300 m AMSL for litter depth and temperature (Table 7).

## Discussion

### Elevational pattern of diversity

This is the first report of the elevational distribution of forest litter ants in the Western Ghats. The observed patterns conform to the mid-elevational rise in species richness and abundance recorded from Philippines (Samson et al. 1997) and Madagascar (Fisher 1996, 1998, 1999a, b, 2002). Although, litter ant communities in the low land forests may support a large proportion of species with narrow elevational range and ecological tolerances (Leston 1978; Samson et al. 1997), a different pattern with the highest ant species richness being specific to the mid-elevation is recorded from Wayanad region.

**Table 2.**
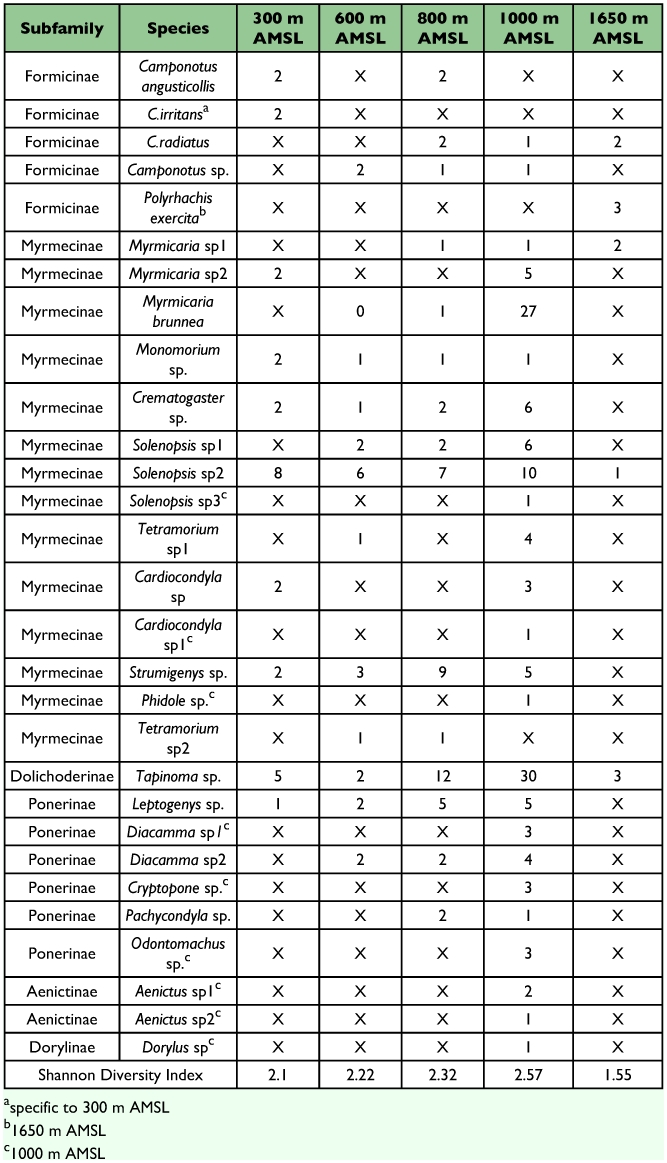
Abundance, richness and diversity of forest litter ant fauna along elevations in the Wayanad region of the Western Ghats.

**Table 3.**
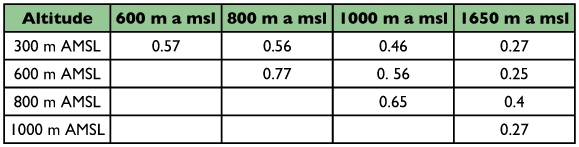
Pair-wise faunal similarity between the forest litter ant assemblages along elevations in the Wayanad region of the Western Ghats.

**Table 4.**
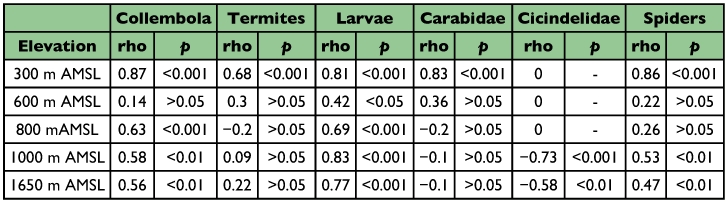
Rank correlation analysis of the relationship between forest litter ants and prey-predator abundance along elevations in the Wayanad region of the Western Ghats.

**Table 5.**
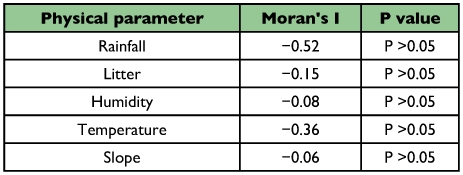
Spatial autocorrelation analysis of physical parameters at five different elevations in the Wayanad region of the Western Ghats.

**Table 6.**
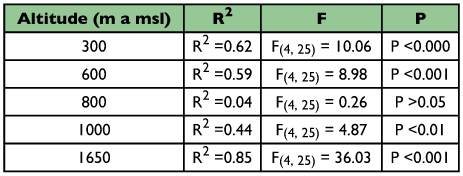
Regression models relating the abundance of forest litter ants to elevation specific physical factors (temperature, litter depth, humidity, and slope of the terrain) in the Wayanad region of the Western Ghats

The mid-elevational peak in ant abundance is attributed to high litter depth, the more open and dry litter habitat conditions and prey resource availability at mid-elevations. It conforms to the observations that ant abundance is higher in more open and dry conditions as moist and humid habitat conditions limits their foraging ability and reduces the time available for foraging on the litter floor (Bruhl et al. 1999). The likelihood of many species from lower and higher elevations overlapping at mid-elevations, which generally provide the most suitable environment for ants (Fisher 1998; Cardelus et al. 2006), and the advantage of being between the more dry forests in the eastern slopes and the moist forests on the west and eastwest slopes, results in having species from both dry and moist forests present that might result in their high abundance in the region.

**Table 7.**
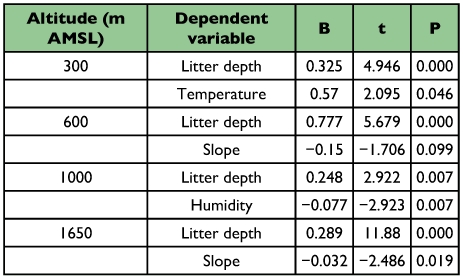
Regression analysis of the influence of physical factors following exclusion of multicollinier variables on forest litter ant abundance along elevations in the Wayanad region of the Western Ghats

High faunal similarity and number of shared species between adjacent elevations (600, 800 and 1000 m AMSL) supports the view that proximity of elevational zones encourages the establishment of marginal populations from adjacent elevations (Fisher 1998). However, given the higher faunal similarity between the adjoining 800 and 600 m AMSL sites than between 800 and 1000 m AMSL sites, we propose that this is related to the presence of ants preferring wet litter habitat conditions at 600 and 800 m AMSL and ants preferring dry litter conditions at 1000 m AMSL sites. Faunal similarity measures suggest a division of the ant assemblage into 3 communities viz., mid-elevation, low elevation and a distinctly different higher elevation assemblage.

Earlier studies of tropical forest litter ant communities found Myrmecinae to be the most common family followed by Ponerinae with a Ponerinae/Myrmecinae ratio of 0.35 to 0.51. (Fisher 1996, 1998). However, an entirely different pattern with the dominance of Myrmecinae followed by Ponerinae in low and mid elevations and dominance of Formicinae followed by Myrmecinae and the absence of Ponerinae in higher elevations was recorded in the present study. It differs from the widely recorded pattern of dominance of Myrmecinae followed by Ponerinae at all elevations in other tropical forest litter ant communities. Further surveys are necessary to establish whether the present litter ant community dominated by Ponerinae is a general characterterists of all stunted montane forests of Western Ghats.

The prevalence of *Tapinoma* and *Solenopsis* at all elevations irrespective of the significant variation in physical and biotic factors indicates that they are the most well adapted ant taxa in the region. Exceptionally high abundance of *Tapinoma* species and *Myrmicaria brunnea* which often tend homopterans for honeydew (Buckley and Gullan 1991; Dejean et al. 2000) at 1000 m AMSL, is a regional pattern possibly linked to the abundance of honey dew-exuding insects in the region. The presence of *Aenictus* species that are specialized on feeding on other ants only at the 1000 m AMSL site conform to the view that ant predators are abundant in ant abundant regions where it is more likely that their preferred prey resource is available (Brühl et al. 1999; Shattock and Barnett 2001). The abundance of arboreal ants, *Polyrhachis* (Watanasit et al. 2003), and the low incidence of true litter ants at 1650 m AMSL may appear, at first sight, as an instance of sampling errors arising from the foraging of arboreal ants on the forest floor closer to sampling sites. However, their prevalence in distant samples suggests that closeness of the canopy to forest floor in the stunted forests at 1650 m AMSL, and the low incidence of litter ants and other predatory fauna excluding Cicindelidae, might lead to frequent ground foraging by arboreal ants in the wet moist litter floor of the stunted montane forests.

Low species richness of true litter ants and cryptic ants (as for example Dacetine ants) in the collections indicate that several forest floor dwelling ants remain unaccounted for. One probable explanation is that since the collections were made soon after the wet monsoon period when the foraging activity of litter ants is lower during wet and cold conditions (Brühl et al. 1998; Olson 1994), many ant species from smaller colonies, and ants present in low number in the sampling area and its surroundings may have been missed. Winkler sampling in warmer weather may lead to more species than in the cold weather and it is necessary to conduct inventories during a warmer period when most species are active (Leponce et al. 2004). Hence, additional sampling in warm summer months is necessary to develop a more complete species list.

### Influence of Biotic and Abiotic factors Biotic factors

Significant positive correlations between prey resources (Collembola and larva) and ants at all elevations indicate that prey resource availability is a strong determinant of ant abundance along elevations of the region. The dominance of termite-preferring *Solenopsis* at the termite abundant 600 m AMSL site and Collembola-preferring *Strumigenys* at the Collembola abundant 800 m AMSL site (Samson et al. 1997; Shattock and Barnett 2001), and the abundance of their respective specific prey resources, conforms to the suggestions from other tropical and neotropical studies (Olson 1994; Brühl et al. 1999) that prey resource availability is important in deciding local ant distribution patterns. Though termites are a major component of tropical forests (Wallwork 1976), their cryptic nature, relative inactivity, range of nesting habits and feeding strategies and patchy distributions within and between habitats results in low sampling efficiency using standard methods (Eggleton and Bignell 1995; Dawes-Gromadski 2005 a, b). The overall low abundance of termites and their insignificant correlation with ants we attribute to the sampling bias arising from low sampling efficiency of Berlese-Tullgren funnel methods to collect termites.

The absence of significant negative correlations between Carabidae and ants, except at lower elevations (300 m AMSL), and the general low abundance of Carabidae at all elevations (Sabu 2005) indicate that Carabidae did not affect the ant abundance in the region. The significant positive correlation between abundance of ants and spiders at a broader regional scale suggest coexistence and prey-resource partitioning by these two groups with similar feeding requirements probably favoured by the general abundance of prey resources. However, lack of correlation between litter ants and spiders at two sites (600 and 800 m AMSL) makes interpretations difficult and indicates that the patterns observed at a broader regional scale are not applicable at local scales. Low abundance of Carabidae and spiders in higher elevations and absence of significant negative correlations with ants, indicate that the often quoted observations (Hölldobler and Wilson 1990; Olson 1994) about probable ecological replacement of ants by spiders and carabids in higher elevations are not applicable in this subtropical study region. However, significant negative correlations between abundance of ants and Cicindelidae indicate the probability of Cicindelidae acting as ecological replacements of ants in the high elevation stunted montane forests.

### Abiotic factors

Litter depth has proved to be the most important predictor of ant abundance at all elevational sites and ant abundance peaked in the mid-elevations with higher litter depth. The predictive ability of all other abiotic variables varied between sites. At the 1000 m AMSL site in mid-elevation in which ants are abundant, humidity is the next best predictor of ant abundance after litter depth. Geographical location of the 1000 m AMSL site on a northern dip in the Wayanad region, separated by several small elevation mountain ranges on the west side that block the moisture-laden southwest monsoon winds sweeping in from the Malabar Coast results in a comparatively warm and dry forest habitat in the region. Consequently, the combined effect of the preference of litter ants towards more open and dry forest floor conditions (Bruhl et al. 1999) and high litter depth and high prey resource availability contributes to the higher ant abundance and species richness in the 1000 m AMSL region. The lack of predictive ability of all physical variables at 800 m AMSL arises from the uniform litter habitat conditions in the region. High ant abundance in the wet evergreen forest site, generally recognised as unfavourable for litter ants, suggests the presence of an assemblage that can withstand extreme environmental conditions such as rain, and high humidity, and low litter temperature in the wet evergreen forests of the Western Ghats (Basu 1997). Earlier studies (Anu and sabu 2006) revealed that the litter ant assemblage at the 800 m AMSL evergreen site consists of a group of taxonomically closely related assemblage more adapted to prevail in moist and wet ecological conditions and is distinct from the ant fauna in the predominantly deciduous forests of the Wayanad region. The presence of two genera, *Acropyga sp* and *Pamtrechina sp* (Shattock and Barnett 2001), that prefer wet rainforests, that have not been recorded from other forest vegetation types in the region (Anu and Sabu 2006), is further evidence for the presence of an ant assemblage adapted to moist wet litter conditions. Litter depth and slope of the terrain are the predictors of ant abundance at higher elevations. Studies elsewhere have shown (Jansen 1973; Olson 1994; Samson et al. 1997; Bruhl et al. 1998) that low litter thickness along with wet moist humid forest floor conditions in higher elevations limits the foraging ability, duration of foraging time and ground nesting by litter ants on the forest floor. The lowest ant abundance and richness at high elevations with steep slopes and strong winds, both of which would not permit litter accumulation, is in full agreement with the above findings. At low elevations, low litter depth and temperature is the best predictor of ant abundance. Though low litter depth is more likely to be linked to rainfall mediated litter runoff in the highly uneven forest floor during monsoon periods, how temperature influences ant abundance in lower elevations is not understood.

## Conclusions

1. Distinct variations in the abundance and richness of leaf litter ants were observed in the forests geographically placed at five elevations. Peak litter ant abundance and richness recorded at mid level elevations in the Wayanad region suggest that these are centres of richest diversity and abundance that should be prioritised as areas for further intense conservation.
2. The Western Ghats consists of a conglomeration of forests lying at various elevations, with distinctive regional patterns in vegetation types and faunal distribution patterns. Hence, the recorded mid-elevation abundance of litter ants in the Wayanad region cannot be taken as a generalization covering the entire Western Ghats. The present study provides a starting point for understanding the distribution pattern of litter ant assemblages and for comparison with altitudinal patterns of other invertebrate taxa in the Western Ghats.
3. A major inference from this study is that the broad patterns observed at a regional level in the Western Ghats may not be always pertinent at local scales, and unless carefully examined will be overlooked in broader regional studies. The present study showed that the influence of physical and biotic factors varies both at the local and regional level, hence further studies of the community structure and diversity of litter ants and other litter fauna along the moist western and dry eastern slopes of Western Ghats should consider the influence of both biotic and abiotic factors both at regional and local levels.

## Abbreviations

AMSL: above mean sea level
